# Clinical Cases

**Published:** 1907-06-22

**Authors:** Edmund Cautley

**Affiliations:** Physic ian to the Belgrave Hospital for Children and the Metropolitan Hospital


### CLINICAL CASES

Shown at the Medical Graduates' College and Polyclinic,

By EDMUND CAUTLEY, M.D., F.R.C.P., Physic ian to the Belgrave Hospital for Children and the Metropolitan Hospital.

### Successful Operation for Perforated Typhoid Ulcer. Fæcal Fistula.

The patient, a girl aged eleven years, was admitted on the seventh day of an attack of typhoid fever. The disease was of a severe type, with high fever, bronchitis, incontinence of urine and fæces, few spots, deafness, and leucopenia. Progress was quite satisfactory until three weeks after admission. The temperature touched the normal level, but incontinence and semi-consciousness persisted. Food was fairly well taken, satisfactorily digested, and there was no special abdominal distension. Suddenly, at 2.10 p.m., she cried out, her expression was anxious, the knees were drawn up, and the hands placed on the abdomen. Breathing was frequent and shallow; pulse small and more frequent; temperature 100° F. There were general distension, tenderness and rigidity of the abdomen, and diminished liver dullness. At 2.30 p.m. there was a leucocytosis of 15,000, a blood pressure (systolic) of 90 mm. and liver dullness was absent. Operation was started at 3.16 p.m. by Mr. C. Gordon Watson, and the last stitch was tied at 3.37 p.m. On making a median incision gas and intestinal contents escaped. A perforation, ¼ inch in diameter and about 18 inches from the ileo-cæcal valve, was quickly found and sewn up; a second very thin ulcer was similarly treated; and an oozing point at the ileo-cæcal valve was secured by a stitch. Patient stood the operation well. On the next day five pints of normal saline were injected slowly into the axillæ. Subsequently to the operation there was a little troublesome vomiting for a few days. The temperature remained normal until the child had a relapse, lasting fourteen days. Unfortunately the median laparotomy wound suppurated and the subjacent gut became adherent to the base and bathed in pus. In this portion of adherent gut softening took place, and there is now a characteristic fæcal fistula, which first began to discharge five weeks ago, twenty-six days after the operation. The child is in other respects quite well.

Hospital cases which perforate in the middle of the day have far the best chance, for the conditions are favourable to immediate diagnosis and operation. Perforation causes about 25-30 per cent. of the deaths. It is more frequent in females than in males in childhood, and vice versa in adults.

Many perforations are only recognised post-mortem, having given no signs during life. Most importance must be attached to pain, increase in pulse and respiration, collapse, and the condition of the abdomen. This child had the typical sudden pain. It may be slight, intense, or paroxysmal, diffuse or localised. Collapse varies in degree with the pain, except when it occurs later from peritonitis. Sometimes there is a rigor or vomiting at the onset. The temperature is of little value. It may rise at first, more often it falls suddenly. The blood pressure sometimes rises, and this is regarded as a differential point in the diagnosis from hæmorrhage. That such a rise does not always occur is clearly shown in this case. Leucocytosis was present, and is of some value; it may be absent. The abdomen is often flat and rigid; at other times distended. Later on peritoneal symptoms develop and the signs of general peritonitis, a complication which may occur independently of perforation. Pain in the abdomen is not always due to perforation, for it may arise from simple colic, constipation, distended bladder, peritonitis, pleurisy, pneumonia, and other rare complications. Acute sudden pain should, however, be always regarded with very grave suspicion.

### Creeping Pneumonia.

This woman is thirty-nine years of age and looks it; but then she has been married twelve years, has five children, is of small physique, and a machinist. All her life she has been delicate. Twelve years ago she had hæmoptysis, and was told her left lung was affected. Since last Christmas she has wasted and suffered from night sweats. A week before admission to hospital she had pain in the side, and was said to have pleuro-pneumonia, with brownish-red sputa for four days. On admission she had signs of pneumonia of the right middle lobe, extending to the upper lobe, and was very ill. Gradually the whole upper lobe became involved. The sputa were nummulated and muco-purulent for two weeks, and the physical signs cleared up but slowly. At first there was continued fever for five days, then it was somewhat hectic in type for about ten days, and then the temperature came down slowly to normal on the eighteenth day after admission, and kept there. The patient still shows a little deficiency of movement, impairment of resonance, and increase of vocal vibrations over the right upper lobe.

